# Water ecological security assessment and spatial autocorrelation analysis of prefectural regions involved in the Yellow River Basin

**DOI:** 10.1038/s41598-022-07656-9

**Published:** 2022-03-24

**Authors:** Meng Qiu, Qiting Zuo, Qingsong Wu, Zhenlong Yang, Jianwei Zhang

**Affiliations:** 1 School of Environmental Science and Engineering, Tianjin University, Tianjin, 300000 China; 2 School of Water Conservancy Engineering, Zhengzhou University, Zhengzhou, 450001 China; 3 Zhengzhou Key Laboratory of Water Resource and Environment, Zhengzhou, 450001 China; 4 College of Humanities and Law, Beijing University of Chemical Technology, Beijing, 100029 China

**Keywords:** Ecosystem ecology, Freshwater ecology, Environmental impact

## Abstract

To have a more comprehensive understanding of the water ecological security status of the Yellow River Basin, this paper constructs a water ecological security evaluation index system founded on the Pressure-State-Response (PSR) model. The indicators are selected by considering factors such as meteorological conditions, population, economy, water resources, water environment, water ecology, land ecology, ecological service functions, pollution control, and capital investment. Then, the “single index quantification-multiple indices syntheses-poly-criteria integration (SMI-P) method was used to determine the water ecological security index (WESI) of 62 cities in the Yellow River Basin, to classify the safety levels, and combined with the spatial autocorrelation analysis to study the regional characteristics. The results prove that: (a) The overall water ecological security of the Yellow River Basin is relatively poor. Half of the 62 cities have reached the second-level warning level, and most of them are concentrated in the upper and middle reaches of the basin. (b) Wetland area is a long-term key factor in the construction of water ecological safety, and the greening rate of built-up areas has an increasing impact on water ecological safety. (c) The overall water ecological security index shows a slow upward trend, with the annual average growth rate was 0.59%. (d) The water ecological security of 62 cities in the Yellow River Basin shows significant spatial autocorrelation. The findings can offer a practical basis for the water ecological management to promote the high-quality development of the Yellow River Basin.

## Introduction

With the increasingly intensified global land conflicts, the overall ecological environment is showing a deteriorating trend. For example, the reduction of natural resources such as forests, water, and grasslands, and the ecological damage caused by the over-exploitation and utilization of natural resources^1^, ecological security is constantly under threat. Among them, the water resources shortage and the pollution of water bodies have made the water ecology increasingly fragile, which has brought risks to the safety of water ecology. In particular, the water ecological security of the river basin is related to the healthy development of the entire river basin. The current situation of water ecology in the Yellow River Basin is not optimistic, with problems such as water shortage, water quality pollution, imbalance of water and sand, and degradation of the water ecosystem. These problems have harmed the economic and social development of the entire basin. Therefore, in-depth analysis and discussion of the current water ecological security situation will lay a good foundation for the watershed’s next step of ecological protection and governance. It is of great significance to the construction of water ecological security and the high-quality development of the watershed.

At present, ecological security issues are highly concerned, and there have been many studies about it. Many scholars have researched the factors affecting ecological security, including climate change^2^, forests and grasslands^3^, rivers and lakes^4^, urban ecosystem^5^, marine ecology^6^, etc. For example, Huang et al.^2^ believed that climate change affects the ecological security index that maintains the stability of the ecosystem. Tolotti et al.^4^ proposed that changes in salinity and turbidity have essential effects on lake ecology. This involves the relevant content of water ecological security.

Water ecological security is a key content in the ecological security system, and it is also an important research content in water security. With the increasing problems of water pollution, eutrophication of water bodies, and lack of water resources, water ecological security has been seriously threatened. The issues have attracted many researchers’ attention. For instance, the examine of Tolotti et al.^4^ showed that the sediment records confirmed the high vulnerability of the water level and salinity changes of Lake Newsiedl. Zhou et al.^7^ founded that the ecological environment water security is lower than domestic water. And agricultural production water, economic and social factors are the key factors affecting rural water security. Some scholars have studied water security from different dimensions. For example, Doeffinger and Hall^8^ analyzed water security from four aspects: the natural scale of phenomena, the scale of data availability, the scale of decision-making, and the precise scale. Monitoring and modeling the water ecological security of large-scale river and lake systems are also the current research focus^9^. Many scholars also evaluate water ecological security based on different methods and models. For example, using driving force-pressure-state-influence-response (DPSIR) model, pressure-state-function-response (PSFR) model, pressure-state -response (PSR) model^10–12^. Cacador et al.^13^ proposed a multi-metric index method based on ecological indicators to evaluate ecological quality. Chen et al.^14^ used the relative risk model (RRM) to evaluate the ecological risks of the water environment. Maggioni et al.^15^ combined physical–chemical and hydro morphological data to evaluate water quality and ecosystems. In summary, most of the research results focusing on water security include water ecological security. In addition, more research has focused on ecological risk assessment and water quality assessment, which are essential content related to water ecological security. However, there are few research results explicitly focusing on water ecological security.

On September 18, 2019, the ecological protection and high-quality development of the Yellow River Basin have become a crucial national strategy^16^. The ecological problem of the Yellow River and its basin has received more and more attention, and many scholars have conducted research from different aspects. For example, Wang et al.^17^ have evaluated the cultivated land resources of the Yellow River Basin in terms of ecological security. Abubaker et al.^18^ have assessed the issues related to drought risk in the Yellow River Basin in terms of hydrological. In addition to assessing arable land, water bodies, and meteorological drought risks, some scholars have also used Analytic Hierarchy Process (AHP) to evaluate soil erosion^19^. In terms of basin evaluation, Zuo et al.^20^ established the Happiness River Index (HRI) evaluation framework, aiming to provide a basis for the ecological environment protection and governance of the Yellow River Basin. Zhou et al.^7^ constructed a “five-in-one” Yellow River comprehensive evaluation system composed of economic, political, cultural, social, and ecological dimensions. Hazbavi et al.^21^ evaluated watershed health from climate and hydrology and constructed a watershed health assessment framework. He et al.^22^ applied the probability density overlapping area method and the joint probability distribution method to assess the ecological risks of heavy metal in the water bodies of various river basins. In terms of basin management, Ratha and Agrawal^23^ developed a watershed management model founded on graph theory to better develop and manage watersheds.

Although there have been numerous studies on Yellow River Basin, most of the findings focus on the general management of the ecological environment of the river basin and the ecological risk assessment of local areas, and few studies have reported on the water ecological security in the whole river basin. Therefore, the main problems related research fields face are as follows: (a) The research objects are mostly concentrated on one aspect of water ecological security, while there is a lack of extensive analysis and evaluation of the water ecological security of the whole river basin. (b) Most of the existing research scales are provinces, and few kinds of research on water ecological security are conducted at the municipal level. (c) Existing research mainly focuses on the research object itself, ignoring the influence factors of the research object.

Combining the questions raised above, we focus on the following aspects: (a) Select essential indicators on the scale of 62 cities in the Yellow River Basin and establish a water ecological security indicator system. (b) Specific analysis of factors affecting water ecological security. (c) Conduct a comprehensive analysis and assessment of the water ecological security status and spatial changes of 62 cities in the Yellow River Basin in the past ten years. The research aims to provide a favorable reference for scientific management of the river basin and promotion of ecological protection as well as promoting the high-quality development of the Yellow River Basin.

The rest of the structure of this paper is as follows: “Methodology” section describes the methods used in this paper, including SMI-P and spatial autocorrelation analysis. “Case study” section expounds the basic facts of the study area and the collection and processing of data. “Result and discussion” section thoroughly analyzes the calculation results from multiple dimensions, comprehensively evaluates the water ecological security, and profoundly analyzes the spatial distribution characteristics and spatial autocorrelation. The main conclusions and deficiencies of this paper are detailed in “Conclusions” section.

## Methodology

### Concept and connotation of water ecological security

The notion of water ecological security is a derivative concept of ecological security. The idea of ecological safety is divided into the overall impact on ecology. The narrow sense refers to the fitness and completeness of the system. It is the degree of protection for human beings in production, life, and health from ecological damage and environmental pollution, including protection basic elements such as food safety, air quality and green environment. Taking the concept of applied ecological security devised by the International Institute of Ecological Analysis (IIASA, 1989), it refers to the aspects of human life, health, well-being, fundamental rights, livelihood security, necessary resources, and the ability of society and humans to respond to environmental changes. The state of non-threatening, including natural ecological security, economic and ecological security, and social-ecological security, constitutes an artificial ecological security system. From the above concepts, it can come to the conclusion that the focus of the idea of ecological security is that the impact or threat of human activities on the ecosystem does not exceed the start-up force of the ecosystem. The ecosystem is the security of the ecosystem, so humans can continue to obtain ecological services and stay in safety status^24^. Through the understanding and definition of ecological security, aquatic ecological security can be defined as a state where the impact of human activities does not cause harm to the aquatic ecosystem. Its connotations include human factors, aquatic biological safety, water resource security, and water environmental security.

### Water ecological security assessment index system

#### Indicator selection

The pressure-state-response model (PSR) is a conceptual model proposed by the Organization for Economic Cooperation and Development (OECD) and the United Nations Environment Programme (UN-EP) and is currently one of the most widely used indicator systems. The model is based on the logical relationship of pressure-state-response, which reflects the mutual effect between humans and the environment. Human activities exert negative effects on the environment, causing the natural environment to change some of the original nature and state. On the contrary, human beings deal with these changes through environmental, economic, and administrative strategies to protect and repair the natural ecological environment.

Choosing appropriate indicators is the first step in the assessment of water ecological security. It is imperative to clarify the connotation of water ecological security and determine the threat factors. A comprehensive analysis of water ecological security requires a quantitative description of the key factors of water ecological security. Water ecological security should be evaluated and analyzed based on the basin’s integrity. Therefore, this paper discussed and selected the evaluation indexes from the macro level and constructed the water ecological security index system from the four dimensions of water ecology, water resources, water environment, and economic society on account of the PSR model.

Based on the pressure-state-response (PSR) framework, this paper collects indicators from the complete and detailed related indicator system literature, including ecological risks, ecological fragility, and watershed ecological security. Then analyze the selected indicators in turn and fit the PSR model. Keep indicators with strong relevance, high fit, and high frequency of use. Finally, following the principles of scientificity, completeness and representativeness, the indicators are further screened to construct a water ecological safety evaluation indicator system, as shown in Table 1.

**Table 1:** Water ecological security evaluation index system.

Target layer	Criterion layer	Indicator layer	Unit	Type
Water ecological safety	Pressure	***A***_***1***_ Annual precipitation	mm	+
		***A***_***2***_ Population density	person/km^2^	−
		***A***_***3***_ Natural population growth rate	‰	−
		***A***_***4***_ Proportion of urban land	%	−
		***A***_***5***_ Proportion of cultivated land	%	−
		***A***_***6***_ Water area	km^2^	+
	State	***B***_***1***_ COD emissions per 10,000 yuan of GDP	kg/ten thousand yuan	−
		***B***_***2***_ Ammonia nitrogen (NH4 + -N) emissions per 10,000 yuan of GDP	kg/ten thousand yuan	−
		***B***_***3***_ NDVI	–	+
		***B***_***4***_ Concentrated drinking water quality compliance rate	%	+
		***B***_***5***_ Water conservation index	%	+
		***B***_***6***_ Proportion of wetland area to total area	%	+
	Response	***C***_***1***_ Stable compliance rate of wastewater discharge from industrial enterprises	%	+
		***C***_***2***_ Centralized treatment rate of urban domestic sewage	%	+
		***C***_***3***_ Green area rate of built-up area	%	+

The pressure layer includes six indicators: annual precipitation, population density, natural population growth rate, Proportion of urban land, Proportion of cultivated land, and water area. Among them, only annual precipitation and water area are positive indicators. It means that the growth of most indicators has brought pressure on the ecological environment, and negatively impacted water ecological security. The state layer also contains six indicators: COD emissions per 10,000 yuan of GDP, ammonia nitrogen (NH4 + -N) emissions per 10,000 yuan of GDP, NDVI, concentrated drinking water quality compliance rate, water conservation index, and proportion of wetland area to total area. Among them, negative indicators are COD emissions per 10,000 yuan of GDP and ammonia nitrogen (NH4 + -N) emissions per 10,000 yuan of GDP. The higher the COD value and ammonia nitrogen emissions, the more organic pollutants in the water, and the more serious the pollution. The response layer includes a stable compliance rate of wastewater discharge from industrial enterprises, centralized treatment rate of urban domestic sewage, and green area rate of built-up area. These three indicators are all positive indicators and play a positive role in protecting and governance of the ecological environment.

#### Development of the indictor standards

The standard classification and standard value determination provided in this article are mainly on account of the following files: (a) The current national recommended standards in the relevant evaluation system and technical guidelines for lake ecological safety investigation and evaluation. (b) National environmental safety assessment report. (c) Corresponding index standards in relevant references. (d) Expert consultation. Water ecological safety standards are divided into five levels, as shown in Table 2.

**Table 2:** Water ecological security status level.

Index interval	Warning level	Risk size
[0, 0.2)	I	Severe warning
[0.2, 0.4)	II	Moderate warning
[0.4, 0.6)	III	Warning
[0.6, 0.8)	IV	Relatively safe
[0.8, 1.0)	V	Safety

### The water ecological security assessment method

#### Single index quantification-multiple indices syntheses-poly-criteria integration(SMI-P)

There are many methods for multi-index evaluation, and the more common ones are the gray comprehensive evaluation method, matter-element analysis method, coupling, coordination method, etc. Based on the in-depth study of the principle of the method and combining the advantages of multiple methods, Zuo^25^ proposed the “single index quantification-multiple indices syntheses-poly-criteria integration” evaluation method (SMI-P). The calculation steps of the method are shown below:

- Single index quantification

The single-index quantitative description adopts the subsection fuzzy membership analysis method. Each index has its harmony degree (SHD) In the index system, which takes the value [0,1]. Since each indicator in the water ecological security system has different properties, a piecewise fuzzy membership function is used to guarantee the dependability of the results. In order to quantify and describe the harmony of a single index, five representative values can be set, namely the worst value (a), poor value (b), passing value (c), better value (d), and optimal value (e), the specific values are 0, 0.3, 0.6, 0.8, 1.

The formula for calculating the harmony degree of the positive index is as follows:

1 $$\mu_{i} = \left\{ {\begin{array}{*{20}l} 0 \hfill & {x_{i} \le a_{i} } \hfill \\ {0.3\left( {\frac{{x_{i} - a_{i} }}{{b_{i} - a_{i} }}} \right)} \hfill & {a_{i} < x_{i} \le b{}_{i}} \hfill \\ {0.3 + 0.3\left( {\frac{{x_{i} - c_{i} }}{{d_{i} - c_{i} }}} \right)} \hfill & {b_{i} < x_{i} \le c_{i} } \hfill \\ {0.6 + 0.2\left( {\frac{{x_{i} - c_{i} }}{{d_{i} - c_{i} }}} \right)} \hfill & {c_{i} < x_{i} \le d_{i} } \hfill \\ {0.8 + 0.2\left( {\frac{{x_{i} - d_{i} }}{{e_{i} - d_{i} }}} \right)} \hfill & {d_{i} < x_{i} \le e_{i} } \hfill \\ 1 \hfill & {e_{i} < x} \hfill \\ \end{array} } \right.\quad \left( {i = 1,2,3, \ldots ,{\text{n}}} \right)$$

The formula for calculating the harmony degree of the reverse index is as follows:

2 $$\mu_{i} = \left\{ {\begin{array}{*{20}l} 1 \hfill & {x_{i} \le e_{i} } \hfill \\ {0.8 + 0.2\left( {\frac{{d_{i} - x_{i} }}{{d_{i} - e_{i} }}} \right)} \hfill & {e_{i} < x_{i} \le d_{i} } \hfill \\ {0.6 + 0.2\left( {\frac{{c_{i} - x_{i} }}{{c_{i} - d_{i} }}} \right)} \hfill & {d_{i} < x_{i} \le c_{i} } \hfill \\ {0.3 + 0.3\left( {\frac{{b_{i} - x_{i} }}{{b_{i} - c_{i} }}} \right)} \hfill & {c_{i} < x_{i} \le b_{i} } \hfill \\ {0.3\left( {\frac{{a_{i} - x_{i} }}{{a_{i} - b_{i} }}} \right)} \hfill & {b_{i} < x_{i} \le a_{i} } \hfill \\ 0 \hfill & {a_{i} < x_{i} } \hfill \\ \end{array} } \right.\quad \left( {i = 1,2,3, \ldots ,{\text{n}}} \right)$$

In the formula, $\mu_{i}$ is the single-index quantitative value of each index. $x_{i} ,a_{i} ,b_{i} ,c_{i} ,d_{i} ,e_{i}$ respectively represent the value, worst value, poor value, passing value, better value, and optimal value of the $i$th index.

(b) Weighted calculation of multiple indicators

The criterion layer contains multiple indicators that can reflect the level of water ecological security. In order to comprehensively consider the impact of indicators on the level of water ecological security and fully obtain the new information brought by the indicator data, a multi-index weighted calculation method is used to obtain the membership of each standard layer. The specific calculation formula is as follows:

3 $$WESI_{t} = \sum\limits_{i = 1}^{n} {w_{i} \mu_{i} }$$

In the formula, $WESI_{t}$ represents the scoring standard of each criterion level. $w_{i}$ is the weight. It is calculated by analytic hierarchy process and entropy method.

(c) Multi-criteria integrated calculation

According to the calculation results of different criterion levels calculated in step (b), the membership degree of each criterion level is weighted to obtain the final water ecological security level.

4 $$WESI = \sum\limits_{t = 1}^{m} {\omega_{t} WESI_{t} }$$

In the formula, $WESI$ is a water ecological safety index. $\omega_{t}$ refers to the weight of the *t*th criterion layer. This paper constructs a pressure-state-response model, and the three criterion layers are equally important for ecological safety evaluation. Therefore, the weights of the three criterion layers are the same as $\omega_{1} = \omega_{2} = \omega_{3} = \frac{1}{3}.$

Refer to the relevant literature^26^ and combine the actual conditions of the Yellow River Basin to finally determine the five feature values of each indicator as shown in Table A.1 of appendix. The average value of each indicator in the 62 prefecture-level administrative regions of the basin is used as the passing value. The optimal value is taken as the highest value and expanded by 10%, and the worst value is taken as the lowest value and reduced by 10%. The better value and the worse value are determined by linear interpolation.

#### Spatial autocorrelation analysis

This paper uses spatial autocorrelation analysis to research the agglomeration and spatial layout characteristics of the water ecological security level of the Yellow River Basin.

Spatial autocorrelation reflects the relevance between a particular geographic phenomenon or a specific attribute value on a regional unit and the same phenomenon or attribute value on a neighboring area unit. It is a metric of the degree of value aggregation in the spatial domain. The measure is Moran’s I, derived from the Pearson correlation coefficient in statistics^27^, to quantify this aggregation property, divided into global spatial autocorrelation and local spatial autocorrelation^28^.

- Global spatial autocorrelation analysis mainly uses Moran’s I to reflect the spatial clustering degree of attribute variables in the entire study area. The application software GeoDa is used for cluster analysis. The calculation formula of Global Moran’s I is:
  5 $$I = \frac{{n\sum\limits_{i = 1}^{n} {\sum\limits_{j = 1}^{n} {\mathop w\nolimits_{ij} \left( {x_{i} - \overline{x} } \right)\left( {x_{j} - \overline{x} } \right)} } }}{{\left( {\sum\limits_{i = 1}^{n} {\sum\limits_{j = 1}^{n} {\mathop w\nolimits_{ij} } } } \right)\sum\limits_{i = 1}^{n} {(x_{i} - \overline{x} )^{2} } }}$$

The formula: n is the number of space observation objects in the study area; $x_{i}$ and $x_{j}$ are the values of the *i*th and *j*th observation counterparts in the space position, respectively. $\overline{x}$ is the average observation value of all objects; $w_{ij}$ is the spatial weight matrix, representing the adjacency relationship between the *i*th and *j*th monitored objects in the spatial position.

The z-score formula of Moran’s I is:

6 $$Z = \frac{I - E(I)}{{\sqrt {Var(I)} }}$$

where *E(I)* represents the expected value of Moran’s I, and *Var(I)* represents the variance of Moran’s I. Moran’s I interval is [− 1,1]. When the value is greater than 0, it indicates a positive spatial correlation between the study areas. When the value is close to 1, there is more substantial spatial autocorrelation. When the value is less than 0, and close to − 1, the negative spatial autocorrelation is more substantial. When the value is close to 0, there is a random distribution.

(b) Use local Moran’s I (also called LISA local spatial autocorrelation index) to reflect the specific accumulation area and spatial aggregation of water ecological security in 62 cities. Local Moran’s I determines the correlation of each spatial unit. For the *i*th area, Moran’s I’s Lisa is defined as follows:
  7 $$I_{i} = \frac{{x_{i} - \overline{x} }}{{S^{2} }}\sum\limits_{j = 1}^{n} {w_{ij} (x_{j} - \overline{x} )}$$

Among them $$i \ne j$$, $$S^{2} = \frac{1}{n}\sum\limits_{i = 1}^{n} {\left( {x_{i} - \overline{x} } \right)}^{2} ,\overline{x} = \frac{1}{n}\sum\limits_{i = 1}^{n} {x_{i} }$$.

Moran’s I’s LISA statistics are tested using z-score:

8 $$Z = \frac{{I_{i} - E(I_{i} )}}{{\sqrt {Var(I_{i} )} }}$$

The LISA coefficient is used to determine whether there is spatial clustering of water ecological security. The LISA coefficient greater than 0 indicates that there is a positive spatial correlation between the local spatial unit and the nearby spatial unit, which is represented by “high-high” or “low-low”; the LISA coefficient less than 0 indicates “low–high” or “high-low”. The performance of the aggregation is negatively correlated.

## Case study

### Study area

**Figure 1:**
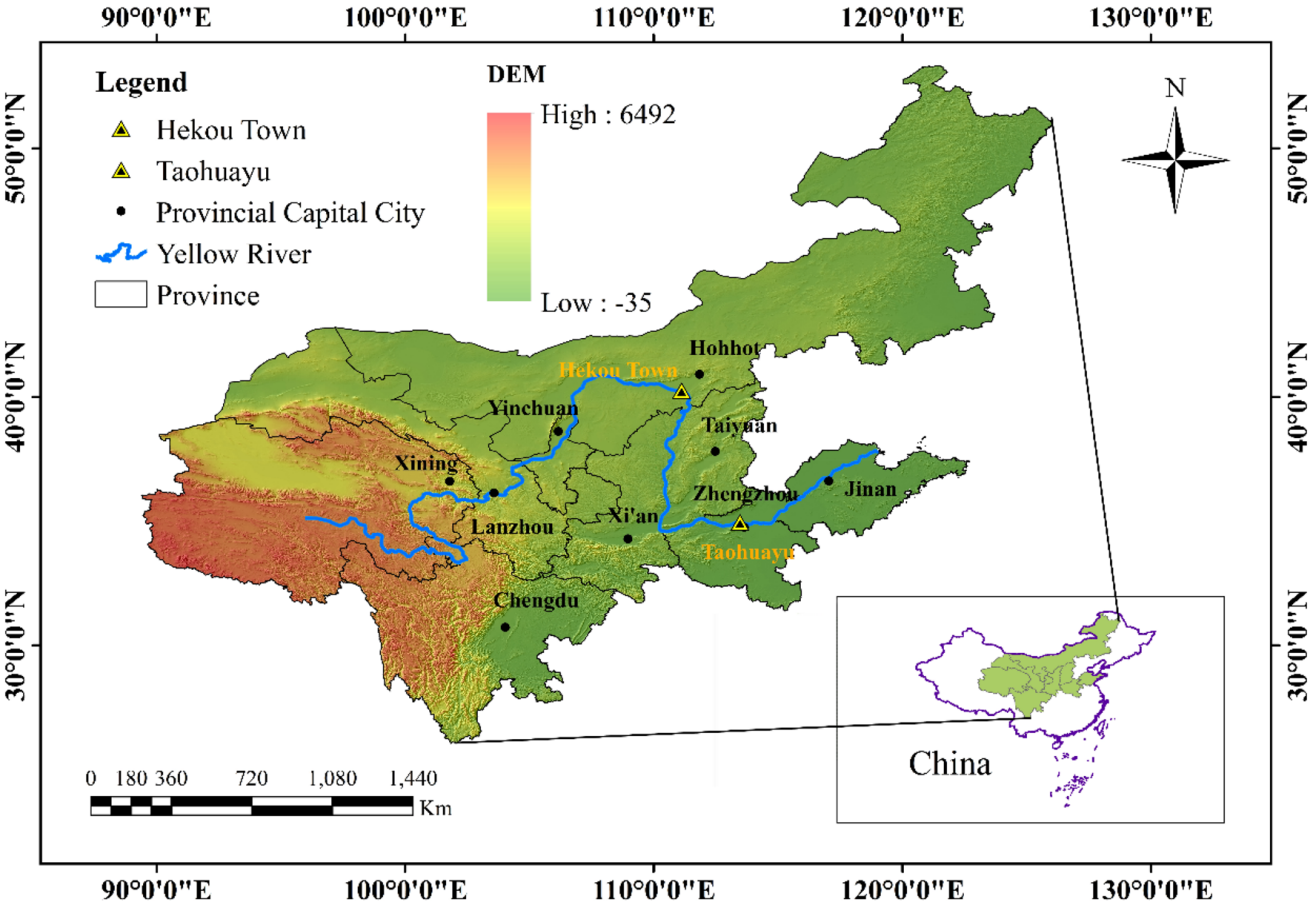
Geographical map of the Yellow River Basin. *Note* This was created by ArcMap-GIS, version 10.5. https://www.esri.com/.

The latitude of the Yellow River is between 34°N and 40°N, and the longitude is between 95°E and 120°E. It is mainly located in the northwest and northern regions of China, backed by the Qinghai-Tibet Plateau and passes through the upper reaches of the mountains and the middle reaches. The Loess Plateau and the North China Plain in the lower reaches of the Bohai Sea flow through provinces including Qinghai, Sichuan, Gansu, Ningxia, Inner Mongolia, Shaanxi, Shanxi, Henan, and Shandong (Fig. 1). The latitude and longitude position and the land and sea position determine that the upper reaches of the Yellow River Basin are dominated by the temperate continental climate, and the middle and lower reaches are dominated by a temperate monsoon climate. The distribution of this climate is the basis for the formation of the hydrological characteristics of the Yellow River. Most of the Yellow River Basin is located in China's central and western regions, where economic and social development is relatively backward. At present, the annual GDP of the Yellow River Basin accounts for only 8% of the national average, and the per capita GDP is about 90% of the national average.

**Figure 2:**
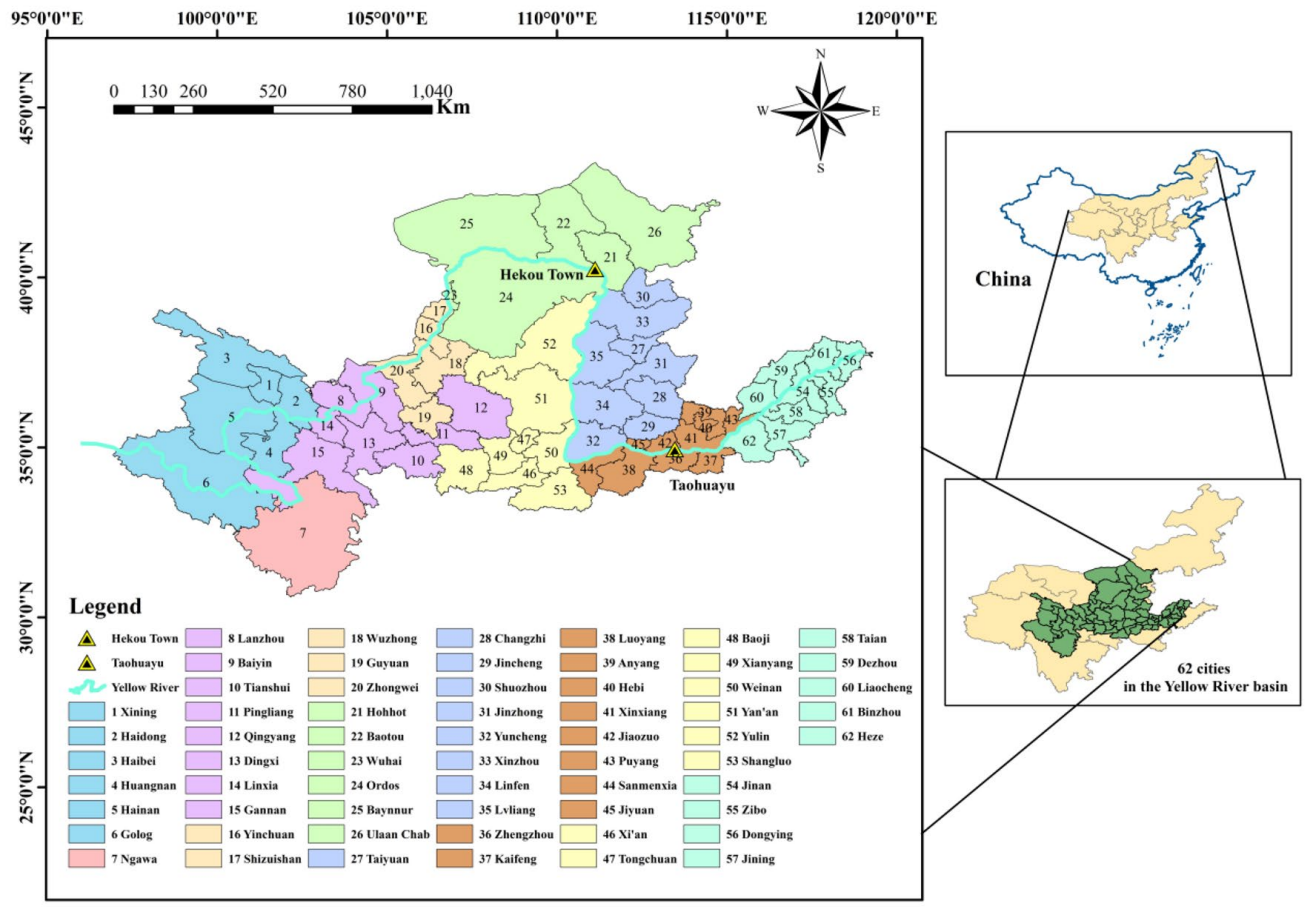
Geographical map of 62 cities in the Yellow River Basin. *Note* This was created by ArcMap-GIS, version 10.5. https://www.esri.com/.

Due to the complex terrain of the Yellow River Basin, the natural environment and the degree of economic development are quite different, so in order to guarantee the comprehensiveness and objectivity of the research, this paper selects 62 cities (Fig. 2) within the Yellow River Basin as the research objects. 62 cities are the main areas involved in the Yellow River Basin, and there are significant differences in natural conditions and economic and social conditions. The study of water ecological security in these cities can objectively reflect the situation of the whole Yellow River Basin. Therefore, it is essential to study water ecological security in 62 cities. Carry out in-depth analysis and discussion on water ecological security to help ecological protection and high-quality development of the Yellow River Basin.

### Data source and description

The following are the data and sources used in this study. The data of indicator *A*_*1*_ Annual precipitation, *A*_*2*_ Population density, *B*_*1*_ COD emissions per 10,000-yuan of GDP, *B*_*2*_ Ammonia nitrogen (NH4 + -N) emissions per 10,000 yuan of GDP, *B*_*4*_ Concentrated drinking water quality compliance rate, *C*_*1*_ Stable compliance rate of wastewater discharge from industrial enterprises, *C*_*2*_ Centralized treatment rate of urban domestic sewage, and *C*_*3*_ Green area rate of built-up area come from the 2009–2019 Urban Statistical Yearbook and Urban–Rural Construction Statistical Yearbook. Among them, *B*_*1*_ is obtained by further processing GDP (100 million yuan) and COD emission (ton) data. *B*_*2*_ is the secondary processing of GDP (100 million yuan) and ammonia nitrogen emission (ton) data.

In addition, from the Resource and Environment Science and Data Center (https://www.resdc.cn/), download land use data and NDVI data with a resolution of 1 km in China, and extract them to 62 prefecture-level cities in the Yellow River Basin using GIS masks for classification extraction Obtained *A*_*4*_ Proportion of urban land, *A*_*5*_ Proportion of cultivated land, *A*_*6*_ Water area, *B*_*3*_ NDVI, *B*_*6*_ Proportion of wetland area to total area; *B*_*5*_ Water conservation index comprehensively considers the proportion of wetland (0.5), grassland proportion (0.15), forest land proportion (0.35) for weighted calculation.

## Result and discussion

### Water ecological security evaluation results of Yellow River Basin

#### Index weight analysis

This study selects the index weights in 2009, 2014, and 2019 for comparative analysis. As shown in Table 3, in terms of space, in the pressure layer, indicator *A*_*6*_ (Water area) has the most prominent weight, and indicator *A*_*3*_ (Natural population growth rate) has the most negligible weight; in the state layer, indicator *B*_*6*_ (Proportion of wetland area to total area) has the most prominent weight, and *B*_*1*_ (COD emissions per 10,000 yuan GDP) has the most negligible weight; in the response layer, indicator *C*_*3*_ (Green area rate of built-up area) has the most prominent weight, and indicator *C2* (Centralized treatment rate of urban domestic sewage) has the most negligible weight. In summary, water area, wetland area, and built-up green space are the key indicators affecting the water ecology of the Yellow River Basin, including natural factors and economic and social factors.

**Table 3:** Water ecological security index weight.

Indicators	Weights		
	2009	2014	2019
*A*_*1*_ Annual precipitation	0.15	0.12	0.13
*A*_*2*_ Population density	0.03	0.03	0.04
*A*_*3*_ Natural population growth rate	0.00	0.02	0.00
*A*_*4*_ Proportion of urban land	0.03	0.04	0.06
*A*_*5*_ Proportion of cultivated land	0.13	0.12	0.11
*A*_*6*_ Water area	0.66	0.66	0.67
*B*_*1*_ COD emissions per 10,000 yuan GDP	0.00	0.00	0.00
*B*_*2*_ Ammonia nitrogen (NH4 + -N) emissions per 10,000 yuan of GDP	0.01	0.01	0.00
*B*_*3*_ NDVI	0.05	0.04	0.03
*B*_*4*_ Concentrated drinking water quality compliance rate	0.01	0.01	0.01
*B*_*5*_ Water conservation index	0.16	0.16	0.15
*B*_*6*_ Proportion of wetland area to total area	0.77	0.79	0.81
*C*_*1*_ Stable compliance rate of wastewater discharge from industrial enterprises	0.38	0.29	0.09
*C*_*2*_ Centralized treatment rate of urban domestic sewage	0.31	0.15	0.01
*C*_*3*_ Green area rate of built-up area	0.31	0.56	0.90

In terms of time, indicators *A*_*6*_ and *B*_*6*_ have equal weights in three years and have always been in an important position. The weight of indicator *C*_*1*_ (the rate of stable compliance of wastewater discharge by industrial enterprises) has fallen for three consecutive years, from 0.38 to 0.09. It shows that after years of environmental management in various cities, the rate of compliance with wastewater discharge standards of industrial enterprises has been continuously increasing. It plays a positive role in the construction of water ecological security. The weight of indicator *C*_*3*_ has increased significantly in three years, from 0.31 in 2009 to 0.90 in 2019, indicating that with the continuous development of urbanization, the built-up area has become larger and larger, which has a massive impact on water ecological security. Therefore, the green area in the built-up area is vital, which is the key to ensuring the urban ecological environment. It is also a critical factor in maintaining the water ecological security.

#### Trend analysis of water ecological security

**Figure 3:**
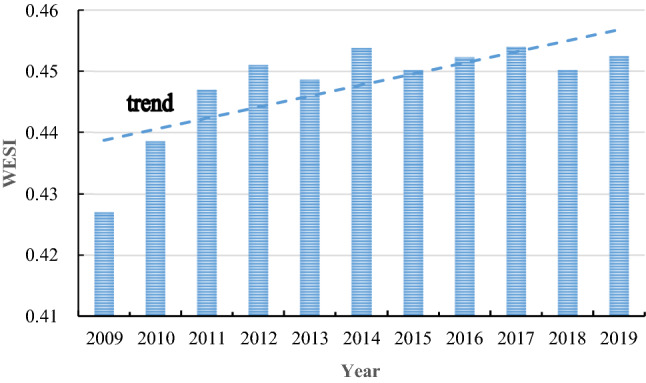
Trend map of water ecological security index (WESI) of nine provinces.

This study is based on Eq. (4) to calculate the WESI of the nine provinces in the past ten years, as shown in Fig. 3. From the perspective of the changes in WESI from 2009 to 2019, the overall trend is slowly increasing. Compared with 2009, WESI increased by 5.96% in 2019, but the average annual growth rate was only 0.59%. The sharp rise stage was in 2009–2012, with an average annual growth rate of 1.84%. Since 2009, there has been no inferior V water in the main stream of the Yellow River, and the water quality has been improving year by year. During this period, the nine provinces implemented the Yellow River Basin Flood Control Plan under the guidance of The State Council. The plan calls for strengthening infrastructure construction in the Yellow River Basin and conducting work such as river improvement and soil and water conservation. Therefore, we will promote the restoration of water ecology in the river basin and improve the safety of water ecology. From 2012 to 2019, WESI showed a trend of ups and downs. This is because the provinces have gradually shifted their development focus to the economy after achieving significant results in restoring water ecology in the river basin. The rapid economic development has brought more significant pressure to environmental governance and hindered water ecological safety improvement.

#### Criterion layer quantitative results

**Figure 4:**
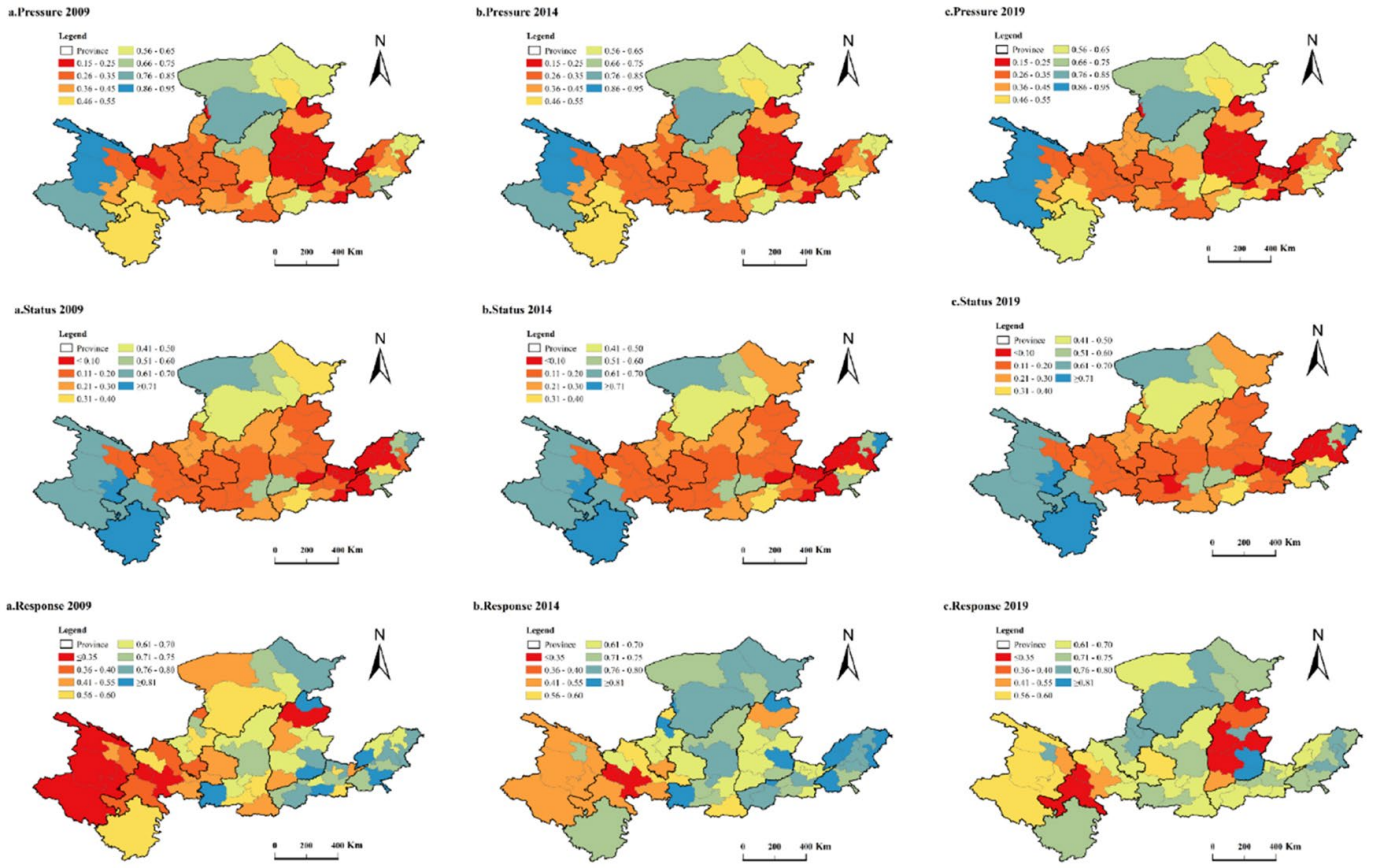
Quantitative spatial distribution map of the 62 cities in the Yellow River Basin. *Note* This was created by ArcMap-GIS, version 10.5. https://www.esri.com/.

To further study and appraise the water ecological security of the study area, this paper quantifies the criteria layers (i.e., pressure, state, response) on account of the SMI-P method. It selects 2009, 2014, and 2019 for comparative analysis. As shown in Fig. 4, the criterion layer has undergone specific changes over time. First of all, the distribution of pressure in 62 cities has not changed much in three years. The areas with more tremendous pressure on water ecological security are mainly concentrated in eastern cities, including Shuozhou, Taiyuan, Jinzhou, Luliang, Linfen, Jincheng, and Changzhi, Anyang, Hebi, Jiaozuo, Puyang, Liaocheng, and other cities. Areas with less pressure are mainly concentrated in western and eastern cities, including Guoluo Tibetan Autonomous Prefecture, Hainan Tibetan Autonomous Prefecture, Haibei Tibetan Autonomous Prefecture, Ordos, Bayannaoer, Yulin, and other cities. In 2009, the precipitation in spring and winter in Lanzhou is less, the degree of drought is serious, and the flood disaster is more severe in flood season, which brings tremendous pressure to the water ecological security. After 2015, Lanzhou continued to implement the Action Plan for Prevention and Control of Water Pollution and then the river chief system was implemented. In 2019, The Work Plan of Lanzhou Municipal Water Pollution Prevention and Control Action in 2019 was issued and implemented. All these measures and actions have laid a foundation for water ecological security. On the contrary, with the rapid development of urbanization and economy and society, the pressure of water ecological security in Jinan has increased.

The larger the value of the status layer, the better the aquatic ecological status. On the contrary, the worse the aquatic ecological security. The overall spatial distribution of the status layer has not changed significantly in the past three years, and the changes are mainly concentrated in some cities. For example, the water ecological security status of Wuhan and Ulan Chab has gradually deteriorated in three years. The reason is that the urban population is becoming denser and sewage discharge is increasing, but related management and measures have not been fully implemented. In Dongying, the water ecological security status improved in 2014 and 2019. According to the Environmental Status Bulletin, in 2014, Dongying deepened its drainage basin pollution control system, continuously strengthened the restraint mechanism to improve river water quality, and carried out a pilot wetland ecological restoration.

In the three years of 2009, 2014, and 2019, the response layer has changed more significantly than the pressure and status layers. It can be seen that the degree of response scarcity has gradually shifted from western cities to eastern cities. The reason can be understood as that due to their superior natural conditions, western cities have relatively weak awareness of water ecological protection and governance, and their ability to respond to emergencies is insufficient. However, with the increasingly prominent ecological and environmental problems, the awareness of maintaining water ecological safety is increasing, and the protection and governance measures are constantly improving. For example, Guoluo Tibetan Autonomous Prefecture, Hainan Tibetan Autonomous Prefecture, and Haibei Tibetan Autonomous Prefecture. Eastern cities are densely populated, urbanization development is faster than western cities, and environmental problems occur more frequently. Therefore, the awareness of ecological and environmental protection is more substantial, the governance system is relatively complete, and responsiveness is relatively good. However, as time progresses, some cities have somewhat slackened their ecological environment governance, and therefore their responsiveness has also weakened. For example, Shuozhou, Jinzhou, Lvliang, Linfen, and other places.

#### Final quantitative results

**Figure 5:**
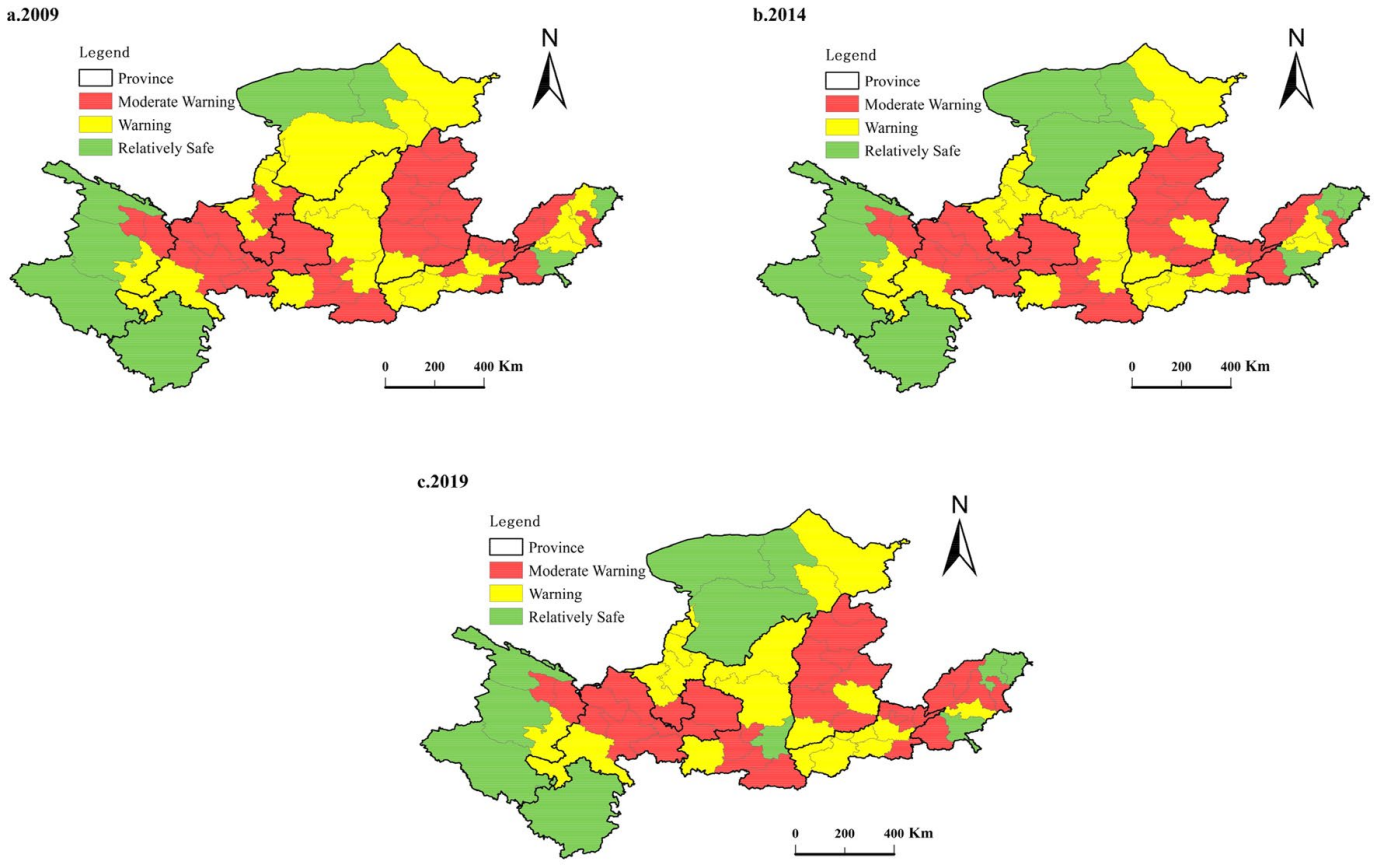
Distribution map of water ecological security status in 62 cities of the Yellow River Basin. *Note* This was created by ArcMap-GIS, version 10.5. https://www.esri.com/.

In order to show the water ecological security status of 62 cities more intuitively, this paper shows the water ecological status level in Table 2 through the GIS spatial distribution map (Fig. 5).

Looking at the overall situation in the past three years, the water ecological security status is relatively stable, with little overall change. The reasons mainly include natural geographical location and economic and social development. In terms of physical geography, the safer areas are concentrated in the upper reaches of the Yellow River Basin, all of which have the characteristics of large land and sparsely populated areas and relatively superior natural conditions. They provide good conditions and foundations for the construction of water ecological security. The moderate warning cities are primarily located in the Loess Plateau and the North China Plain, where water resources are scarce, and the dense population, posing a threat to water ecological security. In terms of economic and social development, relatively safe areas are located in remote areas with inconvenient transportation. The region is dominated by agriculture and animal husbandry, with relatively backward economic development and a low level of urbanization. In addition, the threat to water ecological security is relatively tiny. Residents in the moderate warning area have a significant living demand, and the over-exploitation and utilization of natural resources have led to the destruction of the ecological environment. Therefore, it poses a more significant threat to water ecological security.

Combining Fig. 5 and Table A.2 of appendix, it can be seen that in 2009, there were 8 safer cities, 22 with early warning level, and 32 with moderate warning. Relatively safe cities are concentrated in the southwest and north of the Yellow River Basin; cities with moderate warning level are distributed in the central and eastern areas. In 2014, the number of safer cities increased to 10, and the number of cities with moderate warning level decreased to 30. The means that water ecological security has received more and more attention, and cities have consciously strengthened the protection and governance of water ecology to maintain water ecological security. In 2019, there are 11 relatively safe cities, 21 cities with warning level, and 30 cities with moderate warning level. The overall situation has not changed much, and some cities have changed significantly. For example, Erdos had increased from an early warning status in 2009 to a safer status in 2014, and its safety index has risen from 0.57 to 0.65. Wuzhong has been upgraded from the warning level in 2009 (0.39) to the relatively safe in 2014 (0.44), and the safety index (0.47) in 2019 has also increased. Binzhou had improved from its early warning status (0.60) in 2009 to a relatively safe level (0.64) in 2014, and its safety index (0.66) has also increased in 2019, but the increase is not significant. On the contrary, Jinan has deteriorated from the early warning level in 2009 and 2014 to the moderate warning level in 2019, indicating that the water ecological security of Jinan has been seriously threatened in the process of rapid development.

### Spatial autocorrelation analysis of 62 cities in the Yellow River Basin

#### Global spatial autocorrelation analysis

**Figure 6:**
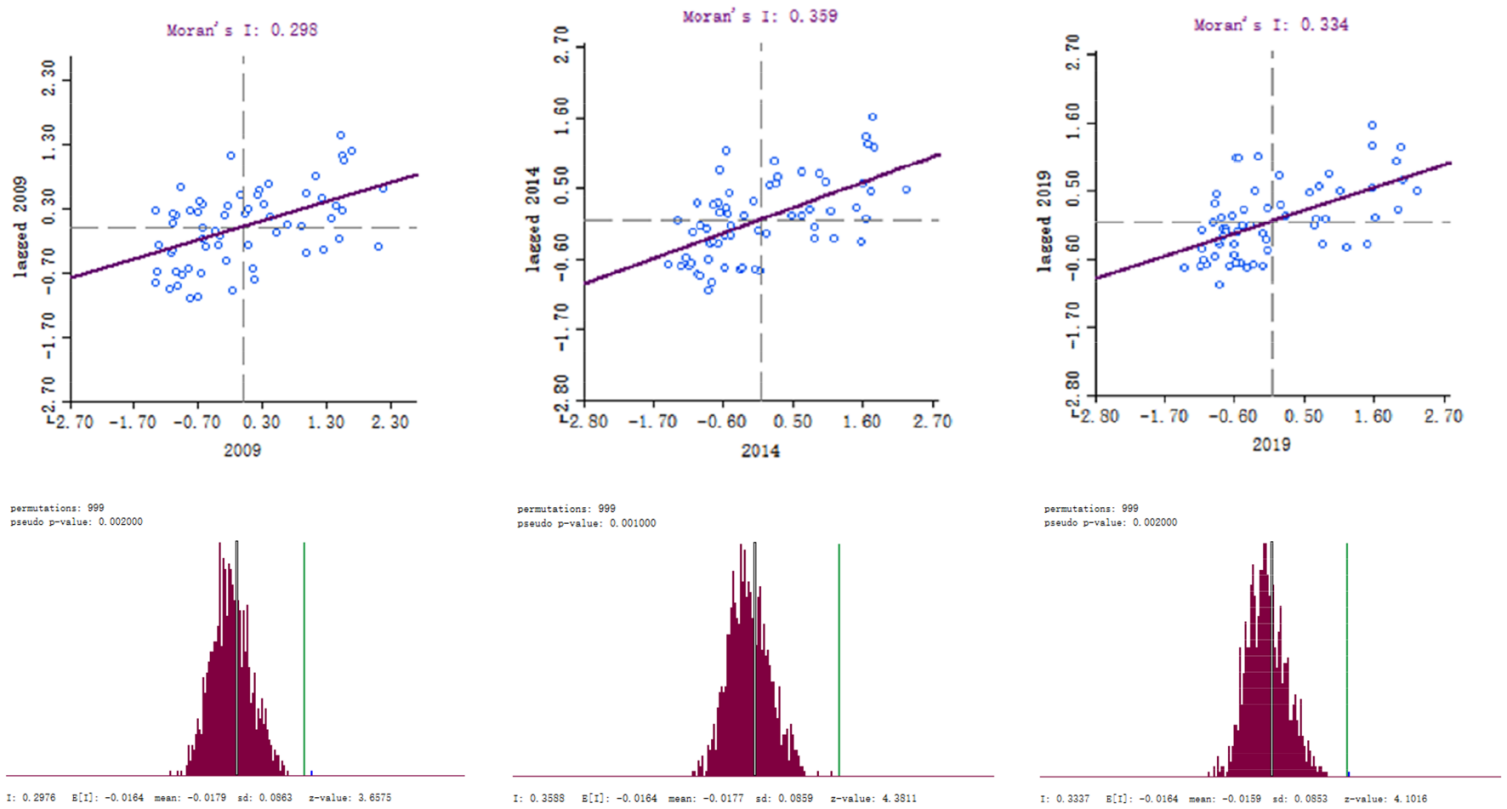
Lisa Scatter diagram and inspection of water ecological security in 62 Cities in 2009, 2014 and 2019.

This paper selects 2009, 2014 and 2019, and analyzes the global spatial autocorrelation based on GeoDa. Combining Table 4 and Fig. 6, the Moran index for these three years was 0.298, 0.359, and 0.334 respectively, which were all in the [0,1] interval, indicating the water ecological security of 62 cities in the past three years showed significant spatial autocorrelation. Moreover, there is a positive spatial correlation, and the spatial autocorrelation is strong. The four quadrants of the scatter chart are high-high (i.e., first quadrant) aggregation area, low–high (i.e., second quadrant) aggregation area, low-low (i.e., third quadrant) aggregation area, and high-low (i.e., fourth quadrant) aggregation area. After testing, z-value > 1.96, *p*-value < 0.05, the study area has significant clustering, proving the strong spatial positive autocorrelation of water ecological security in 62 cities.

**Table 4:** Global Moran’s I and inspection of 62 city water ecological safety.

Year	Moran’s *I*	z-value	*p*-value
2009	0.2976	3.6575	0.002
2014	0.3588	4.3811	0.001
2019	0.3337	4.1016	0.002

Specifically, in 2009, 40 cities were showing positive spatial autocorrelation, accounting for 64.52% of the entire study area. In 2014, 4 more cities were showing positive spatial autocorrelation than in 2009. In 2019, 47 cities showed positive spatial autocorrelation, an increase of 3 from 2014. It shows that the cities showing positive spatial autocorrelation are increasing, but the increase is slight.

#### Local spatial autocorrelation analysis

The global spatial autocorrelation analysis reflects the trend and degree of correlation between different regions in the entire study area. The local spatial autocorrelation analysis can clearly express the concentration and significance of specific areas.

**Figure 7:**
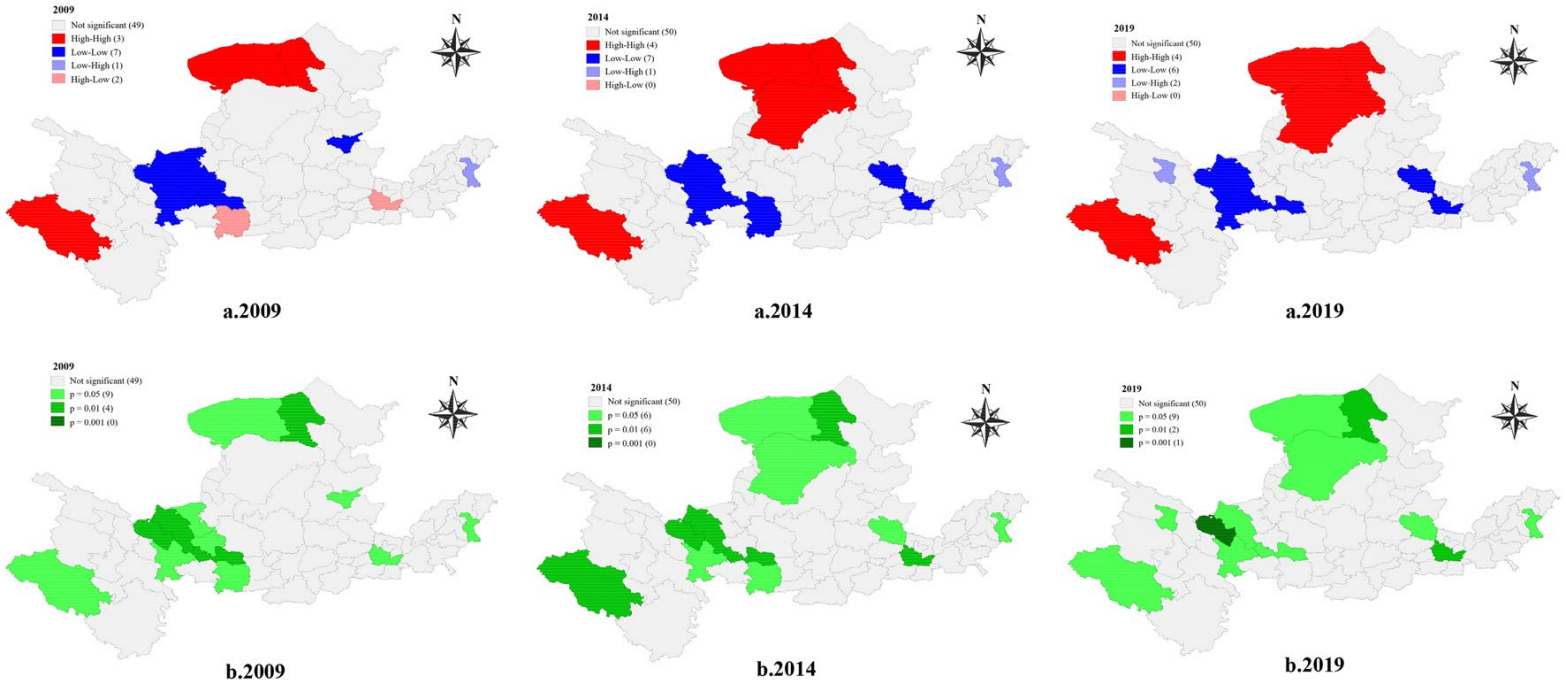
**(a)** LISA cluster map of water ecological safety in 62 cities in 2009, 2014, and 2019, (**b)** LISA significance map of water ecological security of 62 cities in 2009, 2014, and 2019. *Note* This was created by Geoda, version 1.18.0. https://geodacenter.github.io/.
  In combination with Table 5, it can be seen that the proportions of cities with positive spatial correlation (high-high, low-low) in the entire study area in the past three years are 16.13%, 17.74%, and 16.13%. Cities showing negative spatial correlation (low–high, high-low) accounted for 4.84%, 1.61%, and 3.23%. Comparing the two regions, it can be seen that the proportion of spatially positively correlated regions is higher than that of spatially negatively correlated regions, and the number of high-low agglomeration regions in 2014 and 2019 is 0.

- Target layer analysis
  As shown in the Fig. 7 that the significant areas of high-high and low-low are mainly distributed in the upper reaches of the Yellow River Basin and at the junction of the middle and lower reaches. It shows that water ecological security is more important for cities in the upper reaches and middle and lower reaches cities, and the impact is more significant. The upstream contains the river source area and canyon area, rapids, many lakes, swamps, grass beaches, large water yield, rich water resources; the junction of middle and lower reaches is a place where floods occur frequently, and there are lots of dikes, which has a significant impact on water ecology. Therefore, water ecological security is essential for the border area’s upstream, middle, and lower reaches.

**Table 5:** Local spatial autocorrelation types of water ecological security target layer in 62 cities.

Spatial autocorrelation type	2009		2014		2019	
	Quantity	%	Quantity	%	Quantity	%
High–High	3	4.84	4	6.45	4	6.45
Low–Low	7	11.29	7	11.29	6	9.68
Low–High	1	1.61	1	1.61	2	3.23
High–Low	2	3.23	0	0	0	0
Not significant	49	79.03	50	80.65	50	80.65
Total	62	100.00	62	100.00	62	100.00
- Criterion layer analysis

**Figure 8:**
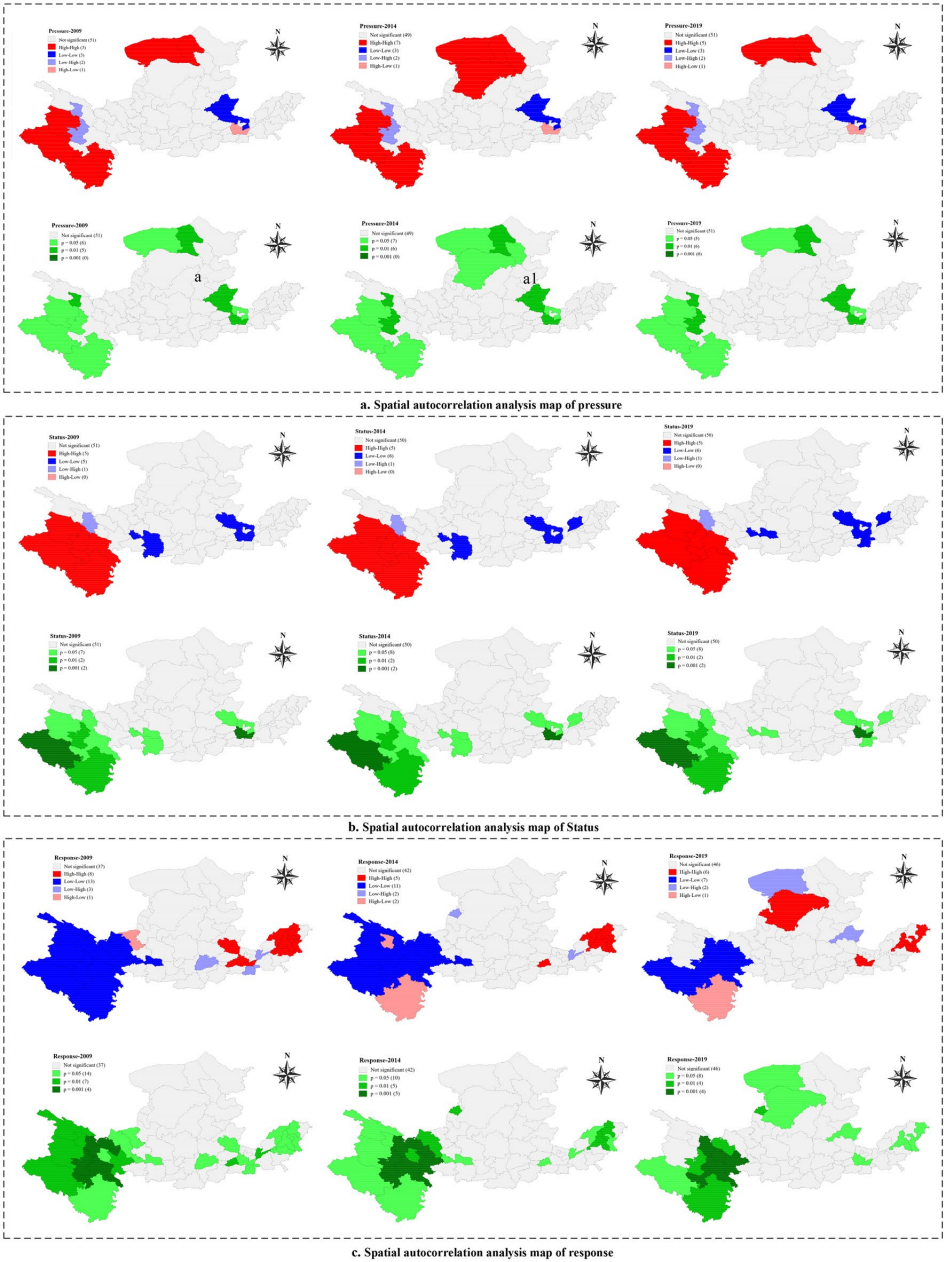
(**a**) Spatial autocorrelation analysis map of pressure, (**b**) Spatial autocorrelation analysis map of state, (**c**) Spatial autocorrelation analysis map of response. *Note* This was created by Geoda, version 1.18.0. https://geodacenter.github.io/.

Pressure, state, and response have varying degrees of impact on the water ecological security of each city, so this article summarizes its characteristics through cluster analysis. In terms of pressure, the overall distribution of the degree of influence by the pressure indicators is stable, and the concentrated areas are in the western, northern, and eastern cities of the Yellow River Basin (Fig. 8a). Cluster distribution and significant changes appeared in very few regions. The main change was that in 2014, Ordos became a high-high area, and the significance of the Huangnan Tibetan Autonomous Prefecture increased.

In terms of state, as shown in Fig. 8b, the concentrated areas are mainly distributed in western and eastern cities, and the main changes are in Baoji, Liaocheng, and Kaifeng. In 2014, Liaocheng was more affected by the status indicators and became more significant. In 2019, the impact of state indicators on Baoji weakened, and the sign changed to insignificant. On the contrary, the influence of status indicators on Kaifeng increased, and its significance increased.

Compared with the pressure and the state, the changes in response are more significant. As shown in Fig. 8c, the concentration area has increased from the west and east to the west, east, and north, and the significance of the concentration area has also changed significantly. In 2009, 21 cities with positive spatial correlation in the study area were distributed in the westernmost and easternmost cities. Among them, 14 cities are highly significant. 4 cities are showing negative correlation, including Zhongwei, Yuncheng, Kaifeng, and Puyang, which are highly significant. In 2014, Aba Tibetan and Qiang Autonomous Prefecture and Xining changed from a positive to a negative correlation, which was very significant. Yuncheng, Kaifeng, etc., have become insignificant. Some areas in the west, such as Haibei and Hainan, have increased significantly, while some areas in the east have decreased significantly, such as Binzhou and Jinan. In 2019, Bayannaoer, Ordos, and Shizuishan were newly concentrated areas in the northern region, which are highly significant and are greatly affected by response indicators. Among them, Ordos and Shizuishan are positively correlated, and Bayannaoer is negatively correlated. At the same time, the number of cities in concentrated areas in the west has decreased, such as Haibei, Hainan, and Xining.

From the above changes, it can be seen that with the continuous development of society and the different needs of ecological environment governance, the attitudes and response intensity of ecological, environmental protection and governance vary from place to place. Therefore, the geographical environment, economy, and population environment are prominent. Cities are more spatially related to stress, state, and response factors.

Combined with Table A.3 of appendix, from an overall point of view, the pressure, state, and response presented in the study area have significant spatial autocorrelation. The proportion of cities with positive correlations presented by the three is more significant than that of negative correlations. Among them, the response layer is more prominent, reaching 33.87%, 25.8%, and 20.97%, respectively, in three years. It can also be seen that the proportion is declining, indicating that with the gradual improvement of environmental problems, some cities have weakened corresponding response measures. And relevant supervision has been relaxed.

## Conclusions

This paper selects 62 cities in the Yellow River Basin for water ecological security assessment and grade zoning. Moreover, Geoda’s spatial autocorrelation analysis of water ecological security clearly understands the spatial change trend of water ecological security in 62 cities. Provide policy basis for further construction of the ecological security barrier of the Yellow River Basin and scientific decision-making. The main conclusions of this paper are as follows:

- The water ecological security of 62 cities is concentrated in three levels: medium early warning, early warning, and relative safety. Relatively safe areas are concentrated in the western and northern parts of the Yellow River Basin. The moderate early warning areas are mainly concentrated in the central and eastern regions of the Yellow River Basin. The rest are warning areas.
- Wetland area is a crucial factor affecting water ecological security for a long time. The impact of the green area rate of built-up area on ecological security increases year by year, and 2019 (0.9) becomes the essential factor.
- The Water Ecological Security Index (WESI) of 62 cities in 2019 increased by 5.96% compared to 2009, but the average annual growth rate was only 0.59%.
- The results of spatial autocorrelation analysis of water ecological security in 62 cities show that the cities in the upper reaches of the Yellow River present a high degree of spatial aggregation and positive spatial correlation, and the water ecological security status is relatively good.
- The water ecological security of some cities to the west, north, and east of the Yellow River Basin is concentrated areas significantly affected by pressure, state and response. The response layer has the most significant changes in time and space, indicating that the relevant management and governance measures and measures adopted by the relevant departments have the most significant impact on the water ecological security of each city.

## Supplementary Information

Supplementary Information.
