# Supplementary material for: Water ecological security assessment and spatial autocorrelation analysis of prefectural regions involved in the Yellow River Basin
Source: Sci Rep. 2022 Mar 24;12:5105. doi: 10.1038/s41598-022-07656-9 (PMC8948252; doi:10.1038/s41598-022-07656-9)
Supplement: Supplementary file 1 — Supplementary Information. [file 41598_2022_7656_MOESM1_ESM.docx]

**Appendix. A**

**Table A.1.** Feature value of evaluation index node

| Indicators | Optimal value | Better value | Passing value | Poor value | Worst value |
| --- | --- | --- | --- | --- | --- |
| *A_1_* | 1,128.16 | 784.48 | 440.80 | 234.22 | 27.63 |
| *A_2_* | 1.90 | 167.26 | 332.63 | 958.52 | 1,584.41 |
| *A_3_* | -17.90 | -5.53 | 6.81 | 24.75 | 42.68 |
| *A_4_* | 0.02 | 3.14 | 6.27 | 16.58 | 26.88 |
| *A_5_* | 0.03 | 20.16 | 40.29 | 66.81 | 93.32 |
| *A_6_* | 3,555.84 | 1,969.69 | 383.54 | 197.05 | 10.56 |
| *B_1_* | 0.06 | 0.63 | 1.20 | 7.68 | 14.16 |
| *B_2_* | 0.01 | 0.06 | 0.12 | 0.66 | 1.20 |
| *B_3_* | 0.96 | 0.80 | 0.65 | 0.40 | 0.14 |
| *B_4_* | 100.00 | 98.13 | 96.26 | 53.98 | 11.70 |
| *B_5_* | 23.25 | 16.74 | 10.23 | 5.12 | 0.01 |
| *B_6_* | 10.08 | 5.27 | 0.46 | 0.23 | 0.00 |
| *C_1_* | 100.00 | 96.85 | 93.70 | 65.13 | 36.57 |
| *C_2_* | 100.00 | 94.14 | 88.27 | 49.17 | 10.06 |
| *C_3_* | 52.05 | 42.52 | 32.99 | 17.22 | 1.44 |

**Table A.2.** Distribution of Water Ecological Security Levels in 62 Cities

|  | 2009 | 2014 | 2019 |
| --- | --- | --- | --- |
| Moderate Warning | Xining, Haidong, Linxia, Dingxi, Lanzhou, Baiyin, Tianshui, Pingliang, Wuzhong, Guyuan, Qingyang, Tongchuan, Shangluo, Xianyang, Xi’an, Shuozhou, Xinzhou, Taiyuan, Lvliang, Linfen, Changzhi, Jinzhong, Jincheng, Jiaozuo, Hebi, Anyang, Puyang, Liaocheng, Dezhou, Kaifeng, Heze, Zibo | Xining, Haidong, Linxia, Dingxi, Lanzhou, Baiyin, Tianshui, Pingliang, Guyuan, Qingyang, Tongchuan, Shangluo, Xianyang, Xi’an, Shuozhou, Xinzhou, Taiyuan, Lvliang, Linfen, Jinzhong, Jincheng, Jiaozuo, Hebi, Anyang, Puyang, Liaocheng, Dezhou, Kaifeng, Heze, Zibo | Xining, Haidong, Linxia, Dingxi, Lanzhou, Baiyin, Tianshui, Pingliang, Guyuan, Qingyang, Tongchuan, Shangluo, Xianyang, Xi’an, Shuozhou, Xinzhou, Taiyuan, Lvliang, Linfen, Jinzhong, Jincheng, Hebi, Anyang, Puyang, Liaocheng, Dezhou, Kaifeng, Heze, Zibo, Jinan |
| Warning | Huangnan, Gannan, Baoji, Zhongwei, Yinchuan, Shizuishan, Wuhai, Ordos, Yulin, Yan’an, Weinan, Yuncheng, Sanmenxia, Luoyang, Jiyuan, Zhengzhou, Xinxiang, Hohhot, Ulaan Chab, Binzhou, Jinan, Tai’an | Huangnan, Gannan, Baoji, Zhongwei, Yinchuan, Shizuishan, Wuhai, Wuzhong, Yulin, Yan’an, Weinan, Yuncheng, Sanmenxia, Luoyang, Jiyuan, Zhengzhou, Xinxiang, Hohhot, Ulaan Chab, Changzhi, Jinan, Tai’an | Huangnan, Gannan, Baoji, Zhongwei, Yinchuan, Shizuishan, Wuhai, Wuzhong, Yulin, Yan’an, Jiaozuo, Yuncheng, Sanmenxia, Luoyang, Jiyuan, Zhengzhou, Xinxiang, Hohhot, Ulaan Chab, Changzhi, Tai’an |
| Relatively Safe | Haibei, Hainan, Golog, Ngawa, Baynnur, Jining, Dongying | Haibei, Hainan, Golog, Ngawa, Baynnur, Jining, Dongying, Baotou, Binzhou, Ordos | Haibei, Hainan, Golog, Ngawa, Baynnur, Jining, Dongying, Baotou, Binzhou, Ordos, Weinan |

**Table A.3.** Local spatial autocorrelation types of water ecological security criterion layer in 62 cities

| Year | Spatial autocorrelation type | High-High | | Low-Low | | Low-High | | High-Low | | Not significant | |
| --- | --- | --- | --- | --- | --- | --- | --- | --- | --- | --- | --- |
|  |  | Quantity | % | Quantity | % | Quantity | % | Quantity | % | Quantity | % |
| 2009 | Pressure | 5 | 8.06 | 3 | 4.84 | 2 | 3.23 | 1 | 1.61 | 51 | 82.26 |
|  | Status | 5 | 8.06 | 5 | 8.06 | 1 | 1.61 | 0 | 0 | 51 | 82.26 |
|  | Response | 8 | 12.90 | 13 | 20.97 | 3 | 4.84 | 1 | 1.61 | 37 | 59.68 |
| 2014 | Pressure | 7 | 11.29 | 3 | 4.84 | 2 | 3.23 | 1 | 1.61 | 49 | 79.03 |
|  | Status | 5 | 8.06 | 6 | 9.68 | 1 | 1.61 | 0 | 0 | 50 | 80.65 |
|  | Response | 5 | 8.06 | 11 | 17.74 | 2 | 3.23 | 2 | 3.23 | 42 | 67.74 |
| 2019 | Pressure | 5 | 8.06 | 3 | 4.84 | 2 | 3.23 | 1 | 1.61 | 51 | 82.26 |
|  | Status | 5 | 8.06 | 6 | 9.68 | 1 | 1.61 | 0 | 0 | 50 | 80.65 |
|  | Response | 6 | 9.68 | 7 | 11.29 | 2 | 3.23 | 1 | 1.61 | 46 | 74.19 |
